# Quantitative photogrammetric methodology for measuring mammalian belly score in the painted dog

**DOI:** 10.1371/journal.pone.0261171

**Published:** 2021-12-14

**Authors:** Gregory Rasmussen, Mari Smultea, Tammy Cloutier, Anthony Giordano, Beth Kaplin, Lisabeth Willey

**Affiliations:** 1 Painted Dog Research Trust, Victoria Falls, Zimbabwe; 2 Smultea Environmental Sciences, Preston, Washington, United States of America; 3 Antioch University New England, Keene, New Hampshire, United States of America; 4 S.P.E.C.I.E.S. – The Society for the Preservation of Endangered Carnivores and their International Ecological Study, Ventura, California, United States of America; 5 University of Rwanda, Kigali, Rwanda; Universitat Autonoma de Barcelona, SPAIN

## Abstract

The use of “belly scoring” can offer a novel, non-invasive objective management tool to gauge food intake between individuals, groups, and populations, and thus, population fitness. As food availability is increasingly affected by predation, ecological competition, climate change, habitat modification, and other human activities, an accurate belly scoring tool can facilitate comparisons among wildlife populations, serving as an early warning indicator of threats to wildlife population health and potential population collapse. In social species, belly scores can also be a tool to understand social behavior and ranking. We developed and applied the first rigorous quantitative photogrammetric methodology to measure belly scores of wild painted dogs (*Lycaon pictus*). Our methodology involves: (1) Rigorous selection of photographs of the dorso/lateral profile of individuals at a right angle to the camera, (2) photogrammetrically measuring belly chord length and “belly drop” in pixels, (3) adjusting belly chord length as a departure from a standardized leg angle, and (4) converting pixel measurements to ratios to eliminate the need to introduce distance from the camera. To highlight a practical application, this belly score method was applied to 631 suitable photographs of 15 painted dog packs that included 186 individuals, all collected between 2004–2015 from allopatric painted dog populations in and around Hwange (n = 462) and Mana Pools National Parks (n = 169) in Zimbabwe. Variation in mean belly scores exhibited a cyclical pattern throughout the year, corresponding to biologically significant patterns to include denning demand and prey availability. Our results show significant differences between belly scores of the two different populations we assessed, thus highlighting food stress in the Hwange population. In the face of growing direct and indirect anthropogenic disturbances, this standardised methodology can provide a rapid, species-specific non-invasive management tool that can be applied across studies to rapidly detect emergent threats.

## Introduction

Regular monitoring of animal populations is critical to facilitate detection of key trends (e.g., demography, survival, and well-being) both within and between populations [1, 2]. This can be done through traditional wildlife monitoring methods such as censuses, but these options are expensive, time consuming, and/or labour intensive [3, 4]. In conjunction with more traditional methods, rapid assessment tools such as body indices may be more cost and time efficient. Body indices are composite measures of changes that serve to rank specific observations. Belly scoring is one of many potential body indices, however, to date, these tools are mostly subjective and have not been used to their full potential [5, 6]. Here we propose using a combination of photogrammetry and body indices to quantitatively assess belly scores (a body index) to monitor individuals and populations, and to facilitate rapid detection of fluctuating parameters that could affect survival.

Photogrammetry has been used to estimate physical dimensions of wild species [7, 8], body condition in terrestrial and marine mammals [9, 10], and nutritional and disease conditions [11, 12]. Photogrammetry and body condition scoring are tools used for both wild and domestic animals to measure, assess, and monitor the well-being of individuals and populations [7, 9, 13–15], but practically, they can also be attained through “citizen science” [16, 17]. For wild or free-ranging animals, accurately measuring body condition generally involves the stress and cost of capture and restraint, which may inhibit the collection of meaningful sample sizes [8, 14]. The use of photographs for body condition assessment decreases this stress and cost, and furthermore, free-standing individuals are more likely to provide a more natural pose than an anaesthetized animal.

Changes in body indices arise as a response to declining ecological and increasing anthropogenic conditions that increase foraging costs, reduce food intake, and result in energetic poverty [18]. These lead to less rapidly measurable indicators to include stress and infectious and non-infectious disease [19]. Collectively, the result will be reduced survival and reproductive success, and ultimately, declining populations [5, 10, 20]. A commonly applied method for large, free-ranging animals is to subjectively score body condition visually based on an ordinal scale [21]. This includes estimating the relative size or distension of an individual’s stomach as a means of assessing fitness level with respect to how full the stomach is, i.e., relative hunger or satiation level [22, 23]. These mostly subjective tools have the disadvantage of being less accurate for evaluating population trends and are subject to interobserver error, a lack of standardised measurement, and variation.

Terminology also varies for visual measuring of belly conditions [24]. Examples of such terms include ‘belly fullness score’ [23], ‘belly size’ [25], and ‘belly score’ [22, 26–28]. These measurement methods were tested on large wild carnivores in the 1970s and 1980s, first on lions (*Panthera leo*) [26], whereby the stomach content volume or mass was visually estimated by viewing the profile of a standing lion. This visual estimation technique to assess food intake on a relative and subjective scale has since been applied to other lion studies [25, 27, 28], cheetahs (*Acinonyx jubatus*) [29, 30], and painted dogs (*Lycaon pictus*) [23].

One shortcoming of subjective visual estimation of belly size to gauge stomach contents is the use of variable scales with no reference to visually specific, discrete morphometric points (points easily identified and distinguished by multiple observers). These subjective visual estimation methods are prone to inconsistent estimates and observer bias, leading to associated criticisms regarding reliability [13, 22]. The use of an algorithm approach (as offered here) provides a structured process for obtaining belly scores based on multiple structural regions of the body on individuals in photographs, introducing inherently less interobserver bias and variability [13], particularly with the assistance of software measuring tools. For example, photogrammetric measurements were reliably used to measure painted dog cranial asymmetry [31] and morphometric differences in painted dog populations across Africa [17], as well as life stages of marbled salamanders (*Ambystoma opacum*) [32] and body dimensions (posture dependent) in primates [33] and leopards [8].

Another challenge of subjective visual estimation of belly size and body condition is the lack of consistency in condition scaling across studies. Examples of scales that have been used are 1 (near starvation) to 14 (kill partially consumed) in cheetahs [30], 1 (as full as possible) to 5 (empty) in lions [25, 26], and 1 (empty) to 4 (belly markedly distended) in painted dogs [23]. In addition, visual methods of estimating belly size use ordinal categorical scales that may mask subtle relationships within the data. Quantitative objective measurement on a continuous scale (i.e., measurements from a photograph determined objectively by software measuring tools) rather than subjective visual categorization offers a more accurate and repeatable method to monitor belly size [13].

Accurate measurements of body condition relative to food consumption levels (and potential nutritional stress [10]) can also be obtained from photographs and eliminates the need to use more invasive and stressful methods [34]. For example, photographs have been used to measure body condition of free-ranging cetaceans, including gray whales (*Eschrichtius robustus*) [35], right whales (*Eubalaena glacialis* and *E*. *australis*) [36], and killer whales (*Orcinus orca*) [37]. These measurements were then equated to nutritional condition/fatness relative to changes in environmental conditions, food availability, and reproductive status. For free-ranging species in forested and remote environments, various photographic methods have been used successfully to assess relative morphometric measurement and analysis. Examples include photographs of sedated wild animals with a reference of known scale (e.g., ruler) included in the photograph [26], and relative assessments of body condition using remotely placed motion-sensor camera traps [14].

Here we present and apply an objective and replicable methodology based on mammalian anatomy that uses defined and easily recognisable anatomical landmarks to quantify relative belly scores in painted dogs. We focus on the distance between the juncture of the floating ribs and those attached to the sternum (i.e., where belly distention starts), as well as the tangents to the lowest point of the belly as discrete measuring points. This allows for the accurate and objective measuring of relative body proportions and ratios of body proportions [13]. These relative measurements are based on the number of pixels and ratios obtained from digital images using software measurement tools, thus eliminating or decreasing subjective and measuring errors. The purpose of this study was both to propose the use of our belly score indices method and provide an example of how this method can be applied to estimate and compare how “well-fed” two painted dog populations were as an indicator of environmental conditions. For example, if one population had significantly or consistently lower belly score indices, what factors might be affecting the prey base or level of food being consumed for that population?

## Methods

### Focal species

The Endangered (IUCN) painted dog is a highly social, cooperatively hunting species with large pack home ranges [38, 39]. Painted dogs are currently limited to isolated and severely reduced populations in sub-Saharan Africa, which persist despite historical campaigns to exterminate them as predatory vermin [32, 40–42]. Remaining populations are primarily scattered across national parks and managed game parks in South Africa, Tanzania, Kenya, Zimbabwe, Zambia, Namibia, Botswana, and Mozambique [41, 43]. Many remaining populations are not considered viable, and as noted in Cameroon, countrywide extirpations occur [44].

Painted dog populations, and those of many other species, are increasingly vulnerable to direct (e.g., bushmeat poaching, vehicular accidents) and indirect (e.g., habitat destruction, food availability, climate change) impacts of human activity. These many threats urgently require effective solutions, including the development of promising rapid assessment tools. The ability to rapidly monitor the health of remnant painted dog populations is essential for the quick detection of changes within and between populations. Systematic and quantitative monitoring of belly score indices is therefore a valuable and practical tool to track the viability of local populations. For instance, a painted dog pack consisting of 8 to 10 adults and yearlings is considered the most efficient, under optimal conditions, at maximizing prey consumption and reproduction relative to their energetic requirements [19, 45]. Information collected via our belly score index method could be used to examine the health and social dynamics associated with equal food sharing and partitioning among pack members [19], or to illustrate differences with other large carnivores that have more hierarchical systems such as lions [26], wolves (*Canis lupus*) [46], and spotted hyenas (*Crocuta crocuta*) [47].

### Study area

**Fig 1:**
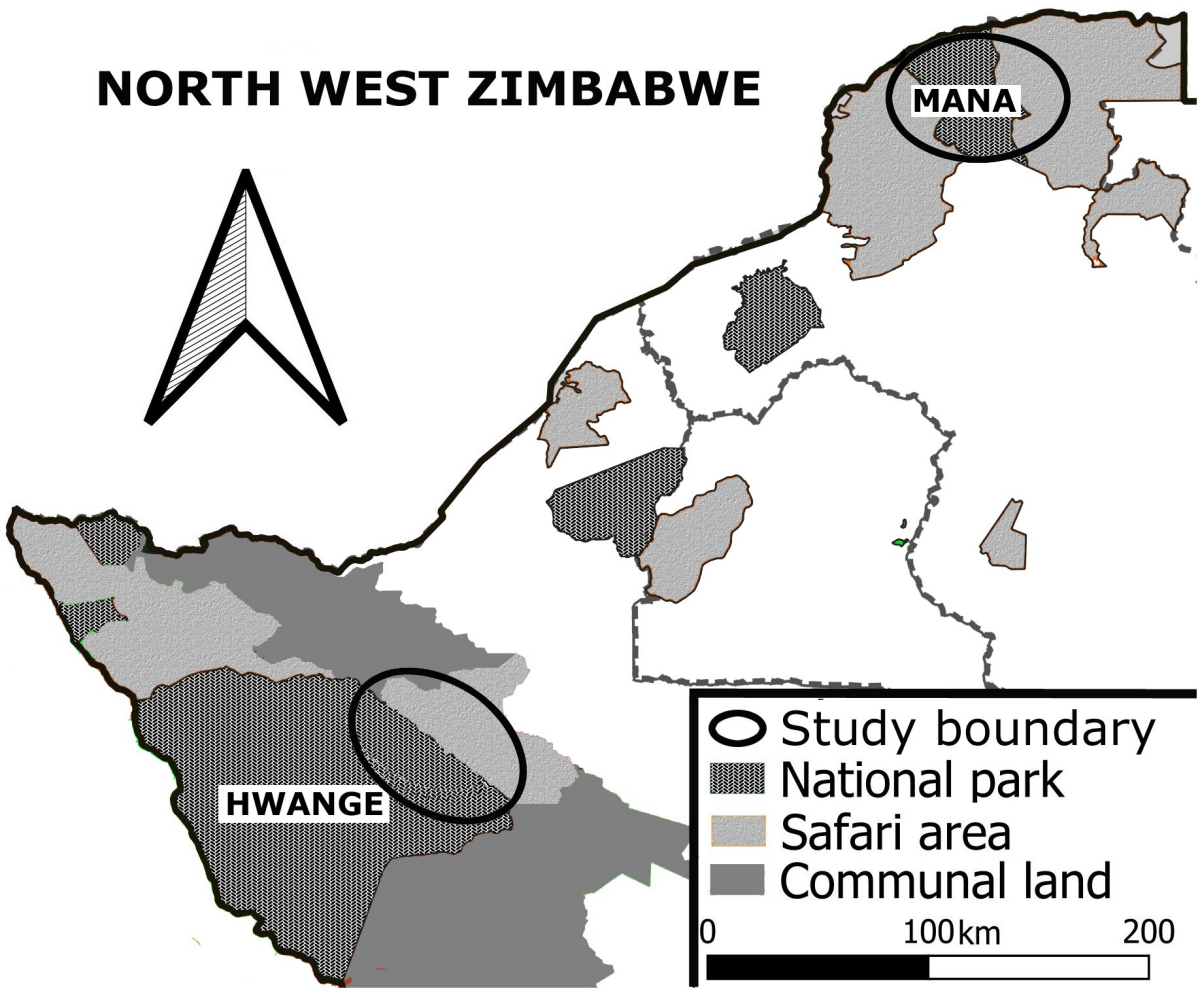
General locations of Hwange National Park and Mana Pools National Park in Zimbabwe.

Our study locations were the eastern portion of Hwange National Park (18°45’S, 27°00’E) and Mana Pools National Park (16°00’S, 29°30’E), and contiguous areas, in northern and western Zimbabwe (hereafter referred to as the Hwange and Mana Pools regions). All research and data collection were conducted under Annual Permit DM 173 issued by the Zimbabwe Department of National Parks and Wildlife Management Authority. No approval by an Institutional Animal Care and Use Committee (IACUC) was required as this was a photographic survey and study, and no handling of animals was required. The two study locations comprised an area of ±4500km^2^ (Fig 1); and the two painted dog populations were parapatrically separated by approximately 430 km. Land bordering Hwange National Park that was utilised by painted dogs was also used for photographic and hunting safaris [17]. The landscape is mainly woodland and scrubland, but also includes savanna and grasslands [48]. African teak (*Baikiaea plurijuga*), acacia (*Acacia spp*.), mopane (*Colophospermum mopane*), bush willows (*Combretum spp*.), and silver cluster-leaf (*Terminaia sericea*) are typical of the primary vegetation [49]. Known prey species of painted dogs in this region include impala (*Aepyceros melampus*), kudu (*Tragelaphus strepsiceros*), and duiker (*Sylvicapra grimmia*) [49]. Other large carnivores inhabiting the region include lions and spotted hyenas, both considered competitors and predators of painted dogs, as well as leopards (*Panthera pardus*) [50].

Similar to Hwange National Park, Mana Pools National Park is adjacent to safari areas that offer photographic and hunting safaris and other recreational activities such as canoeing, fishing, and camping [51, 52], although no hunting is allowed within the park itself [51]. The Zambezi River comprises the northern boundary between Zambia and Zimbabwe. Habitat varies from floodplain with mainly *Faidherbia albida* woodland along the Zambezi River to valley floor *Xylia torreana* dry forests, *Acacia tortillas* woodland savannah, and *Colophospermum mopane* woodlands [51]. Primary painted dog prey species in this area include kudu, impala, eland (*Taurotragus oryx*), and smaller antelope species. Lions, leopards, and spotted hyenas were also present [53].

### Data collection

**Fig 2:**
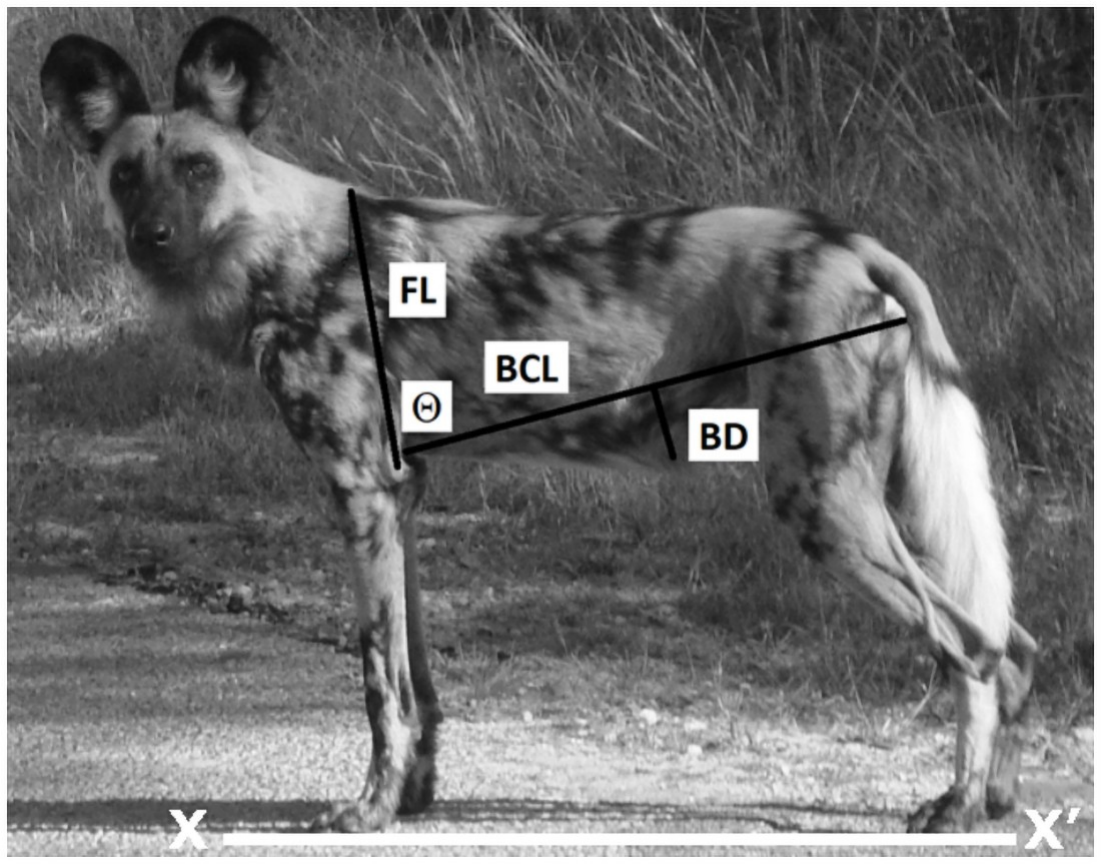
Example of suitable photograph of painted dog (*Lycaon pictus*) showing front leg (FL), belly chord length (BCL), and belly depth (BD) measurements and leg angle (denoted by Θ) used to obtain belly score.

Photographs of painted dogs were collected continuously from 2004–2015 during a long-term behavioural ecology study of the species in the Hwange and Mana Pools regions. We obtained camera-trap images (Stealth Cam model STC-G42NG, STC-G45NG) and photos from HD digital Nikon Coolpix cameras from the Painted Dog Research Trust (PDRT) staff. We also solicited and obtained images from safari operators and tourists. To avoid pseudo-replication, duplicate images of the same dog or pack at the same time event (e.g., within the same AM or PM period) were not used. Data collected included date, time, location (latitude and longitude), group size and composition (e.g., adult, subadult, pup), individual painted dog ID, image number, and photographer/source contact information. Suitable images were defined as those taken of either the right or left lateral side of each painted dog at a right angle (i.e., perpendicular), with the axilla, front leg angle, belly, and sacroiliac process all visible and all four feet aligned on the ground (X-X’ line; Fig 2). The use of ratios and standardisation of the type of photos allowed for analysis enabled us to obtain images from sources “outside” our study as it has been noted that body size and distance from the camera do not influence the accuracy of morphometric measurements [8].

### Image processing

**Fig 3:**
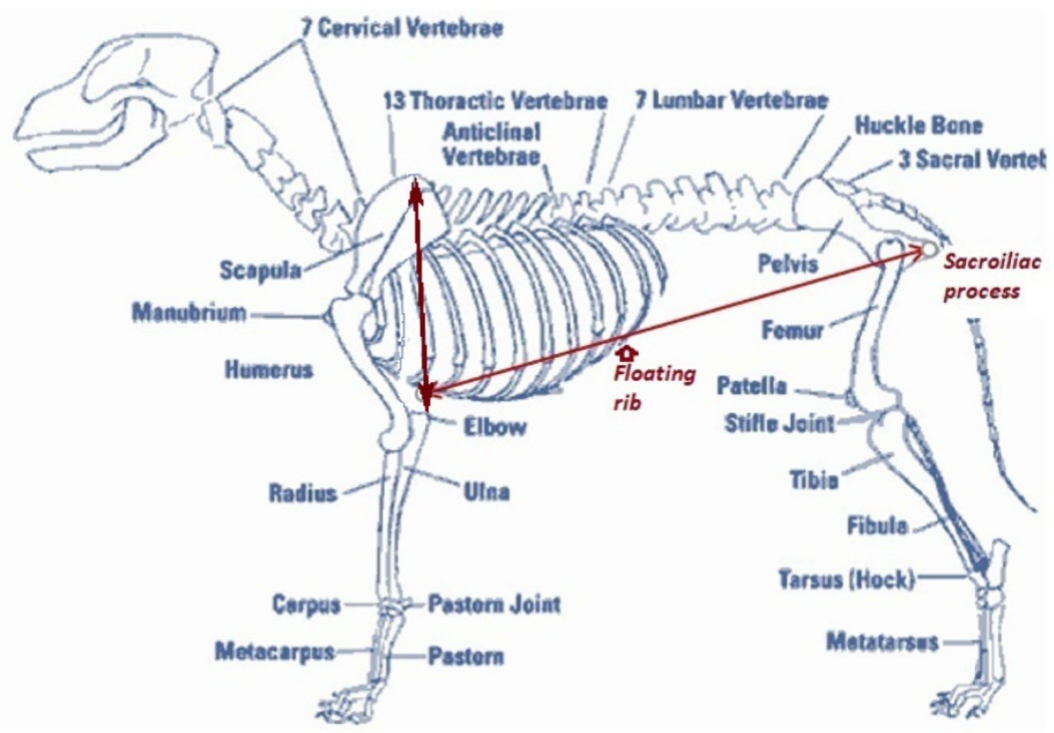
Painted dog (*Lycaon pictus*) skeletal structure demonstrating floating ribs and discrete points from which morphometric measurements to calculate belly score were derived.

Three morphometric measurements based on discrete, easily recognizable morphometric points (Figs 2 and 3) were selected to calculate belly scores; dorsal tip of scapula to lateral epicondyle in the front leg (FL), belly chord length (BCL), and belly drop (BD; Figs 2 and 3; definitions in Table 1). Adobe Photoshop’s measurement tool was used to obtain these three measurements in pixels for all suitable images. S1 Data demonstrates how Photoshop’s (PS) tools are used to identify the discrete morphometric points before measurements are obtained, and S2 Data provides examples of measurements taken from suitable images from both the Hwange and Mana Pools populations. S3 Data provides anonymised data. In addition to recording the pixels for each of the three measurements above, the vertical chord angle (VC) and horizontal chord angle (HC; definitions in Table 1) were documented for each image. These two angle values are also automatically displayed by Adobe when obtaining the FL and BCL pixel measurements. They are used to facilitate finding the angle between the FL and BCL, denoted as Θ.

**Table 1:** Definitions of morphometric terms used to calculate relative belly score for *Lycaon pictus*.

Term	Abbreviation	Definition
Front Leg	FL	Measurement with a line that begins at the highest point of scapula (shoulder blade) and extends to the lateral epicondyle on the elbow
Vertical Chord Angle	VC	Angle of leg obtained when measuring FL from dorsal to ventral points
Belly Cord Length	BCL	Measurement with a line that begins with the protrusion of the sacroiliac process (below the tail) and extends to the axilla (front armpit), following the edge of the floating ribs
Horizontal Chord Angle	HC	Angle of leg obtained when measuring BCL from posterior point (sacroiliac process) to anterior point (armpit)
Belly Drop	BD	Measurement with a line that extends from the discrete point juncture between the sternum-attached and floating ribs to the distal bottom profile of the belly

Here we explain why the VC and HC angles were recorded in addition to the pixel measurements. Using a standardised morphometric photograph that exhibited “good posture” (dorso/lateral profile of individual standing with all four feet touching the ground; Fig 2), we used the 83° leg angle (Θ) as the standard. The deviation from the standard leg angle (Θ minus 83) was denoted as Δ (Table 2). Depending on whether Θ was greater or less than 83, the formula to correct the BCL was: Adjusted BCL = BCL ± FL*tanΔ (Table 2). The Adjusted BCL was used to develop a standardised belly score across different front leg angle postures.

**Table 2:** Formulas used in Excel spreadsheet to derive belly scores for *Lycaon pictus* from morphometric measurements.

Calculation	Formula
Leg angle Θ	Absolute value (HC–VC)
Angular adjustment (degrees) Δ	Leg angle—83
Angular adjustment (radians)	Radians (Leg angle—83)
BCL adjustment (pixels)	Tangent (Angular adjustment radians)*BCL
Adjusted BCL	BCL + BCL adjustment (pixels)
Belly score	Belly drop / Adjusted BCL

Data were entered into an Excel spreadsheet (see example in Table 3), with the associated formulae provided in S4 Data.

**Table 3:** Example of Excel spreadsheet for morphometric measurement data entry. Formulaes to calculate belly scores for *Lycaon pictus* from these measurements are entered into the appropriate cells.

File Name	FL	VC	BCL	HC	Leg angle	Angular adjustment (degrees)	Angular adjustment (radians)	BCL adjustment (pixels)	Adjusted BCL	Belly drop	Belly score
Standardised image, 83° leg angle	335.06	81.10	593.16	164.10	83.00	0.00	0.00	0.00	593.16	90.97	0.1533
Front leg forward (BCL increases)	335.06	83.10	614.80	164.10	81.00	-2.00	-0.03	-21.47	593.33	90.97	0.1533
Front leg backward (BCL decreases)	335.06	79.10	573.13	164.10	85.00	2.00	0.03	20.01	593.14	90.97	0.1533

### Interrater reliability

To test interrater reliability, an intraclass correlation coefficient (ICC; two-way random effects model, absolute agreement, for single observations where both users and measurements were treated as random effects) was calculated using IBM SPSS version 26 [54], α = 0.05. Koo and Yi (2016) [55] stated that there is a lack of acceptable reliability standards for ICC, but noted that at least 30 samples and three raters should be used when possible. The corresponding ICC value scale for such a sample is: poor reliability if values are less than 0.5, moderate reliability for values between 0.5 and 0.75, good reliability for values between 0.75 and 0.90, and excellent reliability if values are greater than 0.90 [55].

For this study, five raters were given a tutorial and then asked to record the FL, BCL, and BD measurements, and corresponding VC and HC angles (Fig 2; definitions in Table 1), for 10 preselected photos of painted dogs. Users identified and highlighted the discrete morphometric points before drawing reference lines and measuring. Although our sample size was smaller than that suggested by [55], we used the proposed value scale to assess our results.

### Data analysis

Belly scores calculated for all suitable images were normally distributed based on the visual assessment of a distribution plot using SPSS. Mixed effects models were run in SPSS to test whether calculated belly scores (the response variable) were related to predictor variables (i.e., region [Hwange or Mana Pools] and month). As some individual dogs were present in multiple images, individual dog ID was used as a random effect. Region (Hwange or Mana Pools) and month were both treated as fixed effects. A t-test revealed there was no effect of sex (i.e., no difference in belly scores between males and females), therefore, the effect of sex was not included in our models.

## Results

A total of 631 photographs of 186 individual dogs from 15 packs were analyzed. Intraclass correlation coefficient was 0.927 (95%, CI = 0.840–0.966), corresponding to “good” to “excellent” reliability (i.e., > 0.75 and 0.90, respectively) on the ICC value scale. The average difference across all users and all three measurements (i.e., FL, BCL, and BD) was 0.18%, and individual differences ranged from 0 to 1.59%.

Belly scores ranged from 0.121 to 0.143 between the two regions (Table 4). We found a significant difference in mean belly scores between the Hwange and Mana Pools populations (F1, 118.0 = 10.721, p = 0.001), as well as significant variation in mean belly scores among months for both populations (F1,139.4 = 8.596, p = 0.004).

**Table 4:** Summary statistics of *Lycaon pictus* belly score means in Hwange and Mana Pool regions, Zimbabwe from suitable photographs obtained from 2004–2015.

Belly score	Mana Pools Region	Hwange Region
Mean	0.138	0.126
Standard error	0.003	0.002
Minimum/Maximum	0.132/0.143	0.121/0.130

**Fig 4:**
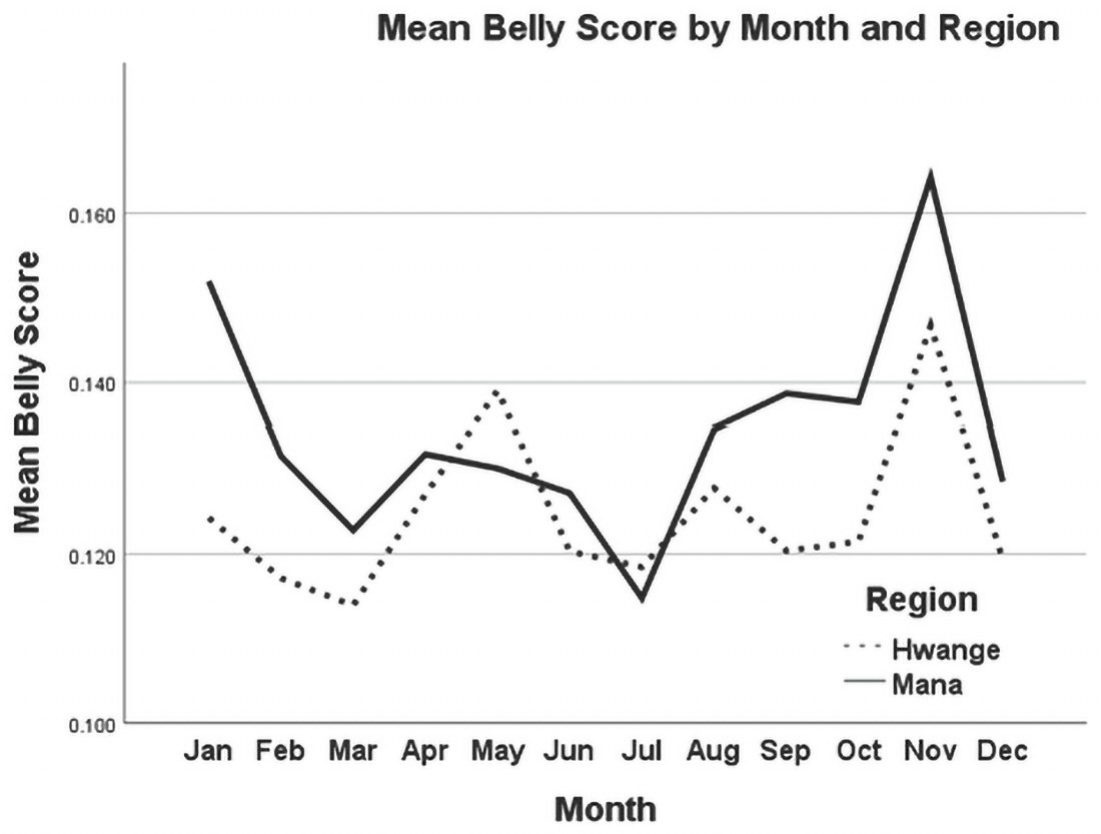
Monthly variation in *Lycaon pictus* belly score means between Hwange and Mana Pools regions, Zimbabwe.

Mean belly scores peaked in April/May and November for individuals in both the Hwange and Mana Pools regions, with smaller peaks in January for both populations (Fig 4). Hwange and Mana Pools groups exhibited similar seasonal variations, and there was no significant interaction between seasonal variation and region (F = 0.629, df = 11.59, p = 0.804). The lowest belly score (corresponding to less stomach distension, which presumably represents reduced food intake and/or high pup demand) occurred during the month of July in Mana Pools; other cyclical lows for that area were evident during March (Fig 4).

## Discussion

Our systematic approach relies on ratios and specific, easily identifiable morphometric measuring points, thereby decreasing error and facilitating more accurate comparisons among individuals both within and among populations. Furthermore, these measurements also yield ecologically relevant information as demonstrated through the similar seasonal patterns exhibited by both populations and the significant differences between the two populations. Previous use of belly scores among wild species has been limited to ordinally-scaled or subjective visual appraisals [21, 25, 26, 29, 30]. Although these studies offered examples of the uses and value of quick assessments, they lacked discrete morphometric reference points and did not account for leg position or angle. These not only introduce ‘noise’ into the data, but reduce precision and repeatability, thus masking finer scale relationships.

Our results highlighted a cyclical pattern in mean belly score throughout the year that corresponds to biologically meaningful patterns; increased belly scores during both prey rutting and lambing/calving seasons, and decreased belly scores (i.e., food or nutritional stress) during the painted dogs’ denning season. For example, the mean belly score was highest during November, the rainy season and peak lambing season for impala and peak calving season for kudu (primary prey species of painted dogs) [49]. In contrast, mean belly scores were lower in May and July, corresponding with the regional denning season for painted dogs and a presumed reduced food intake for individuals due to regurgitating for pups [45].

This is consistent with Giles et al.’s (2014) [24] claim that seasonal variation in body condition is an evolutionary adaptation to survive stochastic, seasonal environments. These results also indicate a decline in belly scores for a total of four months between April and July for the painted dogs in Mana. In Hwange, this decline is only for three months. Research data from both populations highlights that in Mana, painted dogs den earlier, which is consistent with the fact that the timing of denning is temperature related [56]. It is also potentially consistent with the fact that painted dog dens are highly disturbed by both filming and tourism in Mana, but not in Hwange [57], and this would disrupt food procurement, pup feeding rates, and timing of the nomadic phase when they leave the den [19].

Gestation and lactation are energetically costly to many species [36], but particularly to painted dogs [19]. Disturbance of any kind while raising pups may exacerbate these costs. One plausible explanation for the lower belly scores in the Hwange population relative to Mana Pools is that given high baseline reproduction costs, there is further food stress as a result of habitat modifications via elephants. The creation of artificial water holes has facilitated elephant numbers to increase from 1,000 to 44,000 in 70 years [58–60]. In Hwange, this higher relative density has resulted in decreased vegetation and an altered ecosystem structure, which has led to a decrease in both prey numbers and availability [60, 61]. Essential closed woodland habitat, the preferred foraging habitat of painted dogs, has decreased as well [62]. The decrease or loss of both prey and habitat will impose additional energetic and foraging costs [19], thus leading to differences in belly scores.

### Management implications

In the face of declining and disappearing wildlife populations, rapid non-invasive assessment tools such as body indices may offer conservation biologists and protected area managers low-cost monitoring options that are easily replicated. Photogrammetry, as a wildlife/conservation management tool, has been used to: (1) obtain quantitative data on an endangered, potentially nutritionally stressed, killer whale (*Orcinus orca*) population by measuring changes to body condition based on distinctive natural markings [10]; (2) quantify skin disease in giraffes, with shoulder, hip, carpal, and tarsal joints as measuring points [12], and; (3) obtain torso height: horizontal torso length ratios that were noted to have a positive relationship to body condition in free-ranging brown bear (*Ursus arctos*) [9]. Supplementing these studies, our methodology and findings indicate that belly scores can also serve as an important management tool for monitoring free-ranging populations of painted dogs, as well as potentially having practical applications to other carnivore species of concern.

Environmental and nutritional stress data can be time intensive and difficult to obtain [11], however, based on our results and the examples listed above, these types of “rapid assessments” can offer valuable information. Future applications of these management tools may include comparisons among individuals, packs, age groups, reproductive cycles, social structures, and sex between or among seasons and years. In addition, our belly score methodology can assist with assessing general well-being of individuals and populations relative to prey availability, habitat disturbance, human activity, presence or abundance of interspecific competitors, disease or parasitic presence, and current wildlife management practices for both short studies and long-term monitoring efforts. This can be useful in facilitating comparisons in population health among painted dog populations in different regions, and for carnivores in general, by acting as “honest” indicators of foraging success and well-being.

One example where the inclusion of a rapid assessment tool could have been valuable is the painted dog population in Kruger National Park, South Africa. Commencing in 1988, Kruger’s painted dog population was surveyed every five years using photographs of individual dogs collected by tourists [63–65]. From 1988–1995, these 5-year surveys indicated a population that fluctuated between ±400 individuals. Between the 1995 and 2000 surveys however, an estimated 100 individuals were observed [66]. In the absence of more routine monitoring and data driven rapid assessment tools, variation in rainfall was suggested as a potential factor for the steep population decline, but no direct cause was identified [67, 68].

Due to the use of ratios and a standardised criteria to determine photograph suitability for our belly score analysis, data can be collected on a greater scale, potentially offering further insight for wildlife managers and other stakeholders. For example, when performed in the context of citizen science, such as through the evaluation of tourist photos [64], these tools may yield relatively robust data. Our study used over 600 images collected during an 11-year behavioural ecology study. A more concentrated effort that incorporates camera traps (i.e., trail cameras or game cameras) [16], tourists, researchers, and other sources could yield a suitable sample size in a much shorter time frame, as well as contribute valuable data on elusive and endangered species such as painted dogs. Additionally, involving the public not only assists with data collection that may guide management decisions, but also provides an opportunity to educate about pressing conservation and environmental issues; potentially leading to changes in behaviour, advocacy, and community building [16, 17]. Although public inclusion may require additional resources such as time, staff, and funding, rapid assessment tools such as the one we offer here can facilitate increased data collection and allow more information to be more efficiently and rapidly analysed, and the results shared.

## Supporting information

S1 Data Discrete anatomical points used to obtain belly score measurements for *Lycaon pictus* photographs. (TIF)

S2 Data Examples of suitable *Lycaon pictus* images used to obtain measurements for belly score analysis. (PDF)

S3 Data Data set used for *Lycaon pictus* belly score analysis. (PDF)

S4 Data Excel spreadsheet containing associated formulae for *Lycaon pictus* belly score analysis. (XLSX)
