# Supplementary figures and images for: Quantitative photogrammetric methodology for measuring mammalian belly score in the painted dog
Source: PLoS One. 2021 Dec 14;16(12):e0261171. doi: 10.1371/journal.pone.0261171 (PMC8670687; doi:10.1371/journal.pone.0261171)

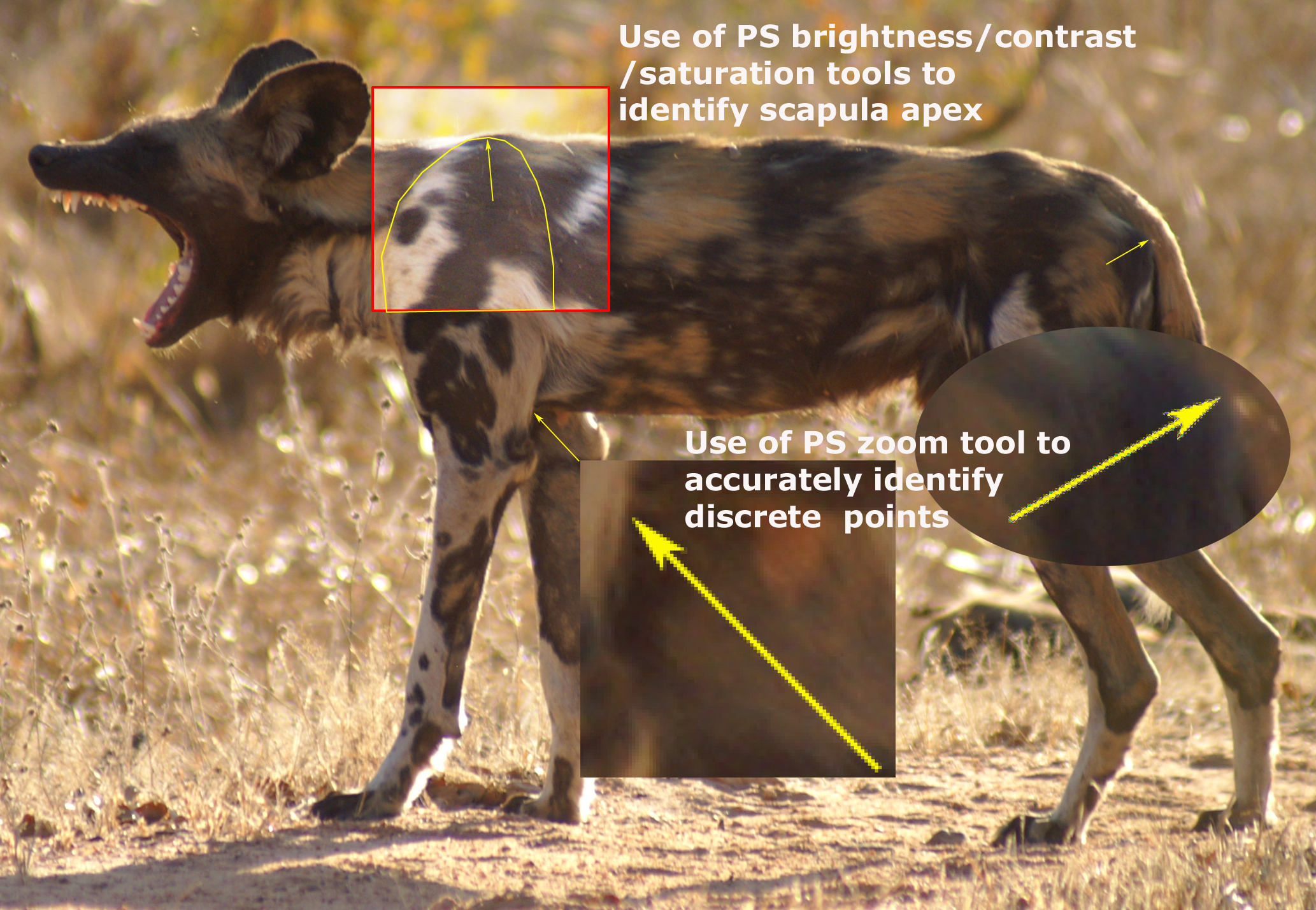

Supplement: S1 Data — (TIF) [file pone.0261171.s001.tif]
