# Supplementary material for: Quantitative photogrammetric methodology for measuring mammalian belly score in the painted dog
Source: PLoS One. 2021 Dec 14;16(12):e0261171. doi: 10.1371/journal.pone.0261171 (PMC8670687; doi:10.1371/journal.pone.0261171)
Supplement: S3 Data — (PDF) [file pone.0261171.s003.pdf]

Individual Dog	region	MONTH	AdjBCLBdrop
Blazey	Hwange	3	0.026689
Owami	Hwange	9	0.0412
Juliet	Hwange	1	0.050112
Inch	Hwange	9	0.050329
Blazey	Hwange	3	0.051147
Eclipse	Hwange	9	0.052696
Argos	Hwange	3	0.05285
Owami	Hwange	12	0.053334
Esther	Hwange	2	0.05389
Juliet	Hwange	1	0.05439
Bullseye	Hwange	2	0.055482
Esther	Hwange	1	0.056655
CrescentMoon	Hwange	9	0.057559
Cyclone	Hwange	9	0.058323
Smokey	Hwange	3	0.060055
Marble	Hwange	9	0.060463
Esther	Hwange	9	0.060846
Esther	Hwange	9	0.061506
Esther	Hwange	9	0.061663
Bullseye	Hwange	12	0.062783
Moth	Hwange	1	0.063178
Annie	Hwange	9	0.063541
Esther	Hwange	9	0.063724
Juliet	Hwange	12	0.064076
Blazey	Hwange	4	0.064734
ShoulderSpot	Hwange	12	0.065129
Prince	Hwange	6	0.065659
CrescentMoon	Hwange	10	0.066088
Antler	Hwange	1	0.066331
Splash	Hwange	9	0.068297
Prince	Hwange	6	0.069214
Papillion	Hwange	3	0.069947
Moth	Hwange	6	0.070224
Amulet	Hwange	9	0.07039
Mango	Hwange	10	0.071431
Khethiwe	Hwange	3	0.07177
Juliet	Hwange	1	0.071877
Osmosis	Hwange	3	0.072219
ShoulderSpot	Hwange	10	0.072309
Owami	Hwange	12	0.072603
Sethule	Hwange	12	0.073574
Blazey	Hwange	2	0.074517
Mango	Hwange	8	0.074585
Esther	Hwange	1	0.075591
Cyclone	Hwange	9	0.075694

Amulet	Hwange	9	0.075716
Spanner	Hwange	6	0.076004
Alpha	Hwange	9	0.076607
Mango	Hwange	9	0.077518
Moth	Hwange	9	0.077533
Esther	Hwange	1	0.078502
Painted	Hwange	3	0.079009
CrescentMoon	Hwange	10	0.079432
Splash	Hwange	9	0.079662
Fob	Hwange	4	0.081201
Bullseye	Hwange	4	0.081318
Scribble	Hwange	9	0.081665
Blazey	Hwange	6	0.081689
Diba	Hwange	11	0.082048
Mango	Hwange	4	0.082316
Cyclone	Hwange	9	0.082472
Margie	Hwange	7	0.082645
Papillion	Hwange	3	0.08295
CrescentMoon	Hwange	9	0.083781
Argos	Hwange	8	0.085084
Jane	Hwange	6	0.085097
Scribble	Hwange	7	0.085341
Moth	Hwange	1	0.085519
Inch	Hwange	6	0.085811
Squirrel	Hwange	4	0.085991
Peepee	Hwange	6	0.086717
Juliet	Hwange	12	0.086837
Sithule	Hwange	12	0.086945
Domms	Hwange	9	0.087779
Uphawu	Hwange	6	0.087928
Blazey	Hwange	2	0.088032
Shoulderspot	Hwange	3	0.088085
Blazey	Hwange	4	0.088427
Owami	Hwange	6	0.088477
Phantom	Hwange	7	0.088704
Spanner	Hwange	9	0.088729
Pita	Hwange	9	0.089048
Blazey	Hwange	2	0.089446
Blazey	Hwange	4	0.089493
Amulet	Hwange	12	0.089932
Sibuyile	Hwange	10	0.089946
Sithule	Hwange	9	0.090273
Pita	Hwange	2	0.090938
CrescentMoon	Hwange	10	0.091274
Owami	Hwange	3	0.091389
Sithule	Hwange	10	0.091742

Alpha	Hwange	2	0.091763
Eclipse	Hwange	9	0.091985
Pita	Hwange	1	0.092409
Kisser	Hwange	4	0.092581
Jim	Hwange	1	0.092713
Temba	Hwange	4	0.092885
Tendai	Hwange	11	0.093121
Pita	Hwange	10	0.093142
Owami	Hwange	3	0.093462
Pula	Hwange	6	0.093668
Burningham	Hwange	5	0.093729
Khethiwe	Hwange	6	0.09398
ShoulderSpot	Hwange	3	0.094418
CrescentMoon	Hwange	7	0.094508
Juliet	Hwange	5	0.094538
Themba	Hwange	10	0.094715
ShoulderSpot	Hwange	12	0.094786
Moth	Hwange	1	0.095371
Phantom	Hwange	2	0.095857
Annie	Hwange	11	0.095917
Blazey	Hwange	3	0.096074
Shoulderspot	Hwange	3	0.096126
Nat	Hwange	3	0.096314
Bullseye	Hwange	4	0.096746
Spanner	Hwange	10	0.097176
Owami	Hwange	9	0.097266
Splash	Hwange	7	0.097835
Smokey	Hwange	9	0.098265
Bullseye	Hwange	6	0.098589
Splash	Hwange	10	0.098976
Tan	Hwange	6	0.099505
Marble	Hwange	9	0.099579
Esther	Hwange	10	0.099666
Marble	Hwange	10	0.10004
Tattoo	Hwange	6	0.100045
Pita	Hwange	6	0.100078
Splash	Hwange	9	0.100161
Cirque	Hwange	3	0.100499
Phantom	Hwange	1	0.100557
Inch	Hwange	9	0.100594
Themba	Hwange	9	0.10063
Chichild	Hwange	9	0.100799
Nat	Hwange	4	0.10102
Painted	Hwange	8	0.101044
Moth	Hwange	3	0.10107
Pita	Hwange	9	0.101269

Beetle	Hwange	1	0.10174
CrescentMoon	Hwange	7	0.101986
Juliet	Hwange	1	0.102113
Esther	Hwange	9	0.102157
CrescentMoon	Hwange	10	0.102301
Circle	Hwange	4	0.10258
Spanner	Hwange	9	0.102851
Papillion	Hwange	2	0.103049
Phantom	Hwange	6	0.10326
Scribble	Hwange	9	0.103394
Themba	Hwange	11	0.103566
Esther	Hwange	11	0.103579
Esther	Hwange	12	0.103912
Amulet	Hwange	4	0.104013
Diba	Hwange	9	0.104391
Splash	Hwange	9	0.104739
Inch	Hwange	10	0.104814
CrescentMoon	Hwange	9	0.104852
CrescentMoon	Hwange	7	0.104939
Moth	Hwange	1	0.105129
Papillion	Hwange	6	0.105301
CrescentMoon	Hwange	7	0.10552
Owami	Hwange	8	0.105568
Painted	Hwange	2	0.105771
Julliet	Hwange	9	0.105779
Osmosis	Hwange	9	0.105872
Sithule	Hwange	9	0.105988
Alpha	Hwange	3	0.106081
Osmosis	Hwange	12	0.106248
Squirrel	Hwange	4	0.106354
Smokey	Hwange	9	0.106388
Smokey	Hwange	9	0.106901
Pupil	Hwange	6	0.107066
Spanner	Hwange	9	0.107143
Scrible	Hwange	10	0.107473
Khethiwe	Hwange	4	0.108006
Sithule	Hwange	12	0.108368
Pita	Hwange	9	0.10838
CrescentMoon	Hwange	9	0.108521
Pita	Hwange	7	0.108934
Moth	Hwange	3	0.10902
Tan	Hwange	5	0.109219
Papillion	Hwange	1	0.109344
Blazey	Hwange	11	0.109565
Nat	Hwange	12	0.109664
Circle	Hwange	3	0.109702

Papillion	Hwange	1	0.110073
Annie	Hwange	1	0.110269
Amulet	Hwange	4	0.110295
Moth	Hwange	12	0.11052
CrescentMoon	Hwange	7	0.111042
Sithule	Hwange	12	0.111418
Temba	Hwange	4	0.111831
Domms	Hwange	8	0.111881
Squirrel	Hwange	1	0.112158
Owami	Hwange	11	0.112208
Spanner	Hwange	7	0.112419
Antler	Hwange	4	0.112517
Domms	Hwange	3	0.112674
Ecko	Hwange	9	0.113216
Owami	Hwange	2	0.113707
Esther	Hwange	3	0.114219
Bullseye	Hwange	12	0.114816
Cyclone	Hwange	9	0.114906
Choppie	Hwange	8	0.115088
Nat	Hwange	1	0.115244
Moth	Hwange	4	0.115276
CrescentMoon	Hwange	7	0.115284
Violin	Hwange	3	0.11552
Choppie	Hwange	3	0.115626
Moth	Hwange	6	0.115683
Juliet	Hwange	5	0.11596
Bullseye	Hwange	1	0.116543
Moth	Hwange	6	0.117365
Sibuyile	Hwange	10	0.117379
Sibuyile	Hwange	10	0.117379
Phantom	Hwange	9	0.11756
Pita	Hwange	10	0.117774
Moth	Hwange	4	0.117868
Spanner	Hwange	7	0.118054
Ecko	Hwange	9	0.118144
HorseShoe	Hwange	9	0.118318
Checkletter	Hwange	9	0.118327
Argos	Hwange	7	0.11833
Teanea	Hwange	4	0.118716
Pita	Hwange	10	0.118724
HorseShoe	Hwange	9	0.119096
Sithule	Hwange	1	0.119132
Vusile	Hwange	9	0.119191
Moth	Hwange	1	0.119192
Juliet	Hwange	1	0.119817
Pita	Hwange	1	0.119863

CrescentMoon	Hwange	7	0.119879
Blazey	Hwange	5	0.119901
Amulet	Hwange	1	0.120054
Kisser	Hwange	2	0.12008
Mango	Hwange	9	0.12069
Bullseye	Hwange	2	0.12091
Juliet	Hwange	12	0.121026
Burningham	Hwange	11	0.121272
Diba	Hwange	3	0.121555
Scribble	Hwange	9	0.121605
Champ	Hwange	3	0.122025
Charles	Hwange	9	0.122178
Scribble	Hwange	9	0.122387
Scribble	Hwange	9	0.122732
Moth	Hwange	8	0.122932
Volcano	Hwange	10	0.123108
Peepee	Hwange	4	0.123406
Pita	Hwange	9	0.123557
Phantom	Hwange	1	0.123788
Papillion	Hwange	1	0.12445
Champ	Hwange	9	0.124457
Burningham	Hwange	9	0.124533
Blazey	Hwange	4	0.124563
Halfmoon	Hwange	1	0.124796
Blazey	Hwange	3	0.125241
Smokey	Hwange	7	0.125412
Esther	Hwange	1	0.125519
Blazey	Hwange	2	0.125653
Painted	Hwange	1	0.125783
Juliet	Hwange	10	0.12623
Staric	Hwange	1	0.126257
Blazey	Hwange	4	0.126349
Nat	Hwange	9	0.126433
Esther	Hwange	1	0.126473
Nat	Hwange	2	0.126863
Pupil	Hwange	5	0.127084
Osmosis	Hwange	7	0.127261
Owami	Hwange	1	0.127679
Blazey	Hwange	4	0.127761
Esther	Hwange	12	0.128326
Spanner	Hwange	10	0.12839
Antler	Hwange	10	0.128428
Pita	Hwange	9	0.12927
Sibuyile	Hwange	9	0.129389
Juliet	Hwange	12	0.129449
ShoulderSpot	Hwange	12	0.129858

Tattoo	Hwange	4	0.130178
ShoulderSpot	Hwange	9	0.130198
Esther	Hwange	4	0.130306
Unknown	Hwange	8	0.130377
Sibuyile	Hwange	9	0.130651
Beans	Hwange	7	0.130668
Owami	Hwange	3	0.130698
Choppie	Hwange	7	0.130978
Owami	Hwange	1	0.131365
Horseshoe	Hwange	8	0.131863
Fob	Hwange	6	0.132046
Blezey	Hwange	6	0.132221
Vusile	Hwange	10	0.133282
Smokey	Hwange	9	0.133477
Papillion	Hwange	1	0.134109
CrescentMoon	Hwange	9	0.134368
Bullseye	Hwange	11	0.134662
Esther	Hwange	1	0.13492
Pita	Hwange	9	0.135105
Circle	Hwange	3	0.135147
Checkletter	Hwange	5	0.135453
Bullseye	Hwange	12	0.135574
Scribble	Hwange	9	0.135739
Bullseye	Hwange	6	0.135969
Chichild	Hwange	1	0.135979
Charles	Hwange	6	0.137103
Vusile	Hwange	9	0.137234
Mango	Hwange	8	0.137359
Solo	Hwange	1	0.137439
Prince	Hwange	9	0.137599
Kisser	Hwange	5	0.137823
Beans	Hwange	9	0.137832
Juliet	Hwange	9	0.138135
Esther	Hwange	9	0.138153
Sithule	Hwange	1	0.138216
Turtle	Hwange	10	0.138292
Khethiwe	Hwange	9	0.138438
CrescentMoon	Hwange	9	0.138543
Bullseye	Hwange	4	0.138579
Scribble	Hwange	10	0.138766
Brush	Hwange	4	0.138819
Spanner	Hwange	10	0.139041
Esther	Hwange	2	0.139085
Champ	Hwange	6	0.139653
Annie	Hwange	9	0.139722
ShoulderSpot	Hwange	10	0.140198

Papillion	Hwange	10	0.140229
Spanner	Hwange	9	0.14043
Bracket	Hwange	6	0.140491
Papillion	Hwange	10	0.140902
ShoulderSpot	Hwange	9	0.1413
Cyclone	Hwange	10	0.141562
Moth	Hwange	12	0.142033
Prince	Hwange	8	0.142418
Marble	Hwange	6	0.143414
Sithule	Hwange	2	0.143521
Chichild	Hwange	3	0.143837
Sithule	Hwange	1	0.144101
Twiggy	Hwange	8	0.144314
Burningham	Hwange	6	0.144325
Scribble	Hwange	9	0.144353
Esther	Hwange	4	0.1446
Jane	Hwange	12	0.145588
Charles	Hwange	3	0.145914
Chichild	Hwange	3	0.145921
Alpha	Hwange	1	0.146112
Cirque	Hwange	4	0.146402
Cirque	Hwange	11	0.146649
Juliet	Hwange	1	0.146713
Pula	Hwange	4	0.146757
Blazey	Hwange	12	0.146875
Bullseye	Hwange	1	0.147203
Hacket	Hwange	7	0.147279
Sithule	Hwange	1	0.14773
Mango	Hwange	3	0.148121
Juliet	Hwange	9	0.148144
Sophie	Hwange	1	0.148304
Moth	Hwange	3	0.148309
Chichild	Hwange	4	0.148315
Circle	Hwange	4	0.148774
Owami	Hwange	9	0.149024
Squirrel	Hwange	1	0.150152
Amulet	Hwange	9	0.150201
Painted	Hwange	10	0.151248
Pita	Hwange	9	0.151752
Jane	Hwange	3	0.151813
CrescentMoon	Hwange	7	0.152005
Beatie	Hwange	5	0.152228
Mango	Hwange	3	0.152303
Scribble	Hwange	9	0.152423
HorseShoe	Hwange	9	0.15243
Trace	Hwange	7	0.152605

ShoulderSpot	Hwange	2	0.15267
Squirrel	Hwange	2	0.152888
Bullseye	Hwange	1	0.152943
Cirque	Hwange	1	0.153192
Jim	Hwange	6	0.153211
Annie	Hwange	3	0.153871
Scribble	Hwange	7	0.154002
Scrible	Hwange	10	0.154155
Staric	Hwange	5	0.154318
CrescentMoon	Hwange	10	0.154454
Pupa	Hwange	6	0.154936
Sithule	Hwange	1	0.154949
Sibuyie	Hwange	9	0.155102
Vusile	Hwange	11	0.15525
Browny	Hwange	4	0.155325
Owami	Hwange	1	0.155357
Prince	Hwange	6	0.155496
Spanner	Hwange	10	0.155664
Blazey	Hwange	12	0.156288
Jane	Hwange	6	0.15696
Smokey	Hwange	7	0.157045
ShoulderSpot	Hwange	2	0.157449
Squirrel	Hwange	1	0.157661
Amulet	Hwange	9	0.157869
Bullseye	Hwange	11	0.158281
Pita	Hwange	9	0.159153
CrescentMoon	Hwange	12	0.159321
Bullseye	Hwange	12	0.15996
Blazey	Hwange	2	0.160503
Khethiwe	Hwange	9	0.160653
Rhombus	Hwange	6	0.160841
Bullseye	Hwange	5	0.160992
Papillion	Hwange	2	0.161336
Painted	Hwange	6	0.16147
Choppie	Hwange	4	0.162304
Splash	Hwange	3	0.163177
Peepee	Hwange	11	0.164687
Saddle	Hwange	3	0.164783
Jane	Hwange	8	0.164819
Squirrel	Hwange	1	0.165387
HorseShoe	Hwange	8	0.165412
Mango	Hwange	9	0.166272
Squirrel	Hwange	1	0.166543
Valance	Hwange	1	0.166564
Vusile	Hwange	9	0.166907
Jim	Hwange	5	0.167151

Splash	Hwange	9	0.167233
Temba	Hwange	6	0.1673
Osmosis	Hwange	3	0.168581
Temba	Hwange	6	0.168688
Blazey	Hwange	6	0.169132
Checkletter	Hwange	4	0.170234
Kisser	Hwange	2	0.170814
Pula	Hwange	11	0.171368
Alpha	Hwange	9	0.171818
Moth	Hwange	12	0.172592
Argos	Hwange	9	0.173002
Prince	Hwange	11	0.1734
Chichild	Hwange	4	0.174417
Halfmoon	Hwange	3	0.175418
Squirrel	Hwange	11	0.175795
Jim	Hwange	9	0.177137
Sithule	Hwange	9	0.178182
Bullseye	Hwange	12	0.180902
Chichild	Hwange	12	0.181127
Teanea	Hwange	4	0.182691
Ring	Hwange	4	0.18282
Cyclone	Hwange	10	0.18298
Scribble	Hwange	9	0.183361
Checkletter	Hwange	8	0.184012
Squirrel	Hwange	1	0.185296
Pupa	Hwange	5	0.186761
Prince	Hwange	9	0.187105
Bullseye	Hwange	11	0.187928
Smokey	Hwange	10	0.188521
Cyclone	Hwange	9	0.189658
Charles	Hwange	4	0.189933
Champ	Hwange	4	0.191582
Jane	Hwange	5	0.19163
Osmosis	Hwange	1	0.19173
Sibuyile	Hwange	9	0.191763
Squirrel	Hwange	1	0.193209
Hacket	Hwange	3	0.195544
Jane	Hwange	9	0.196763
Smokey	Hwange	10	0.199777
Sithule	Hwange	1	0.202044
Jane	Hwange	9	0.203238
Papillion	Hwange	6	0.207393
Splash	Hwange	9	0.209486
Antler	Hwange	9	0.210202
Vusile	Hwange	9	0.211594
Squirrel	Hwange	12	0.215057

Chichild	Hwange	4	0.215229
Pupil	Hwange	11	0.247018
Pupa	Hwange	11	0.251202
Ace	Mana	8	0.068806
Qint	Mana	8	0.069116
Coby	Mana	9	0.072385
Manta	Mana	7	0.075544
Vundu	Mana	9	0.077619
Ace	Mana	7	0.081909
Ecko	Mana	6	0.082934
Fergy	Mana	7	0.083332
Collar	Mana	9	0.083853
Hockey	Mana	8	0.084357
Female2	Mana	9	0.084844
Ace	Mana	8	0.085593
Cirrus	Mana	8	0.086809
Trident	Mana	9	0.08817
Bow	Mana	9	0.090627
8**	Mana	10	0.0913
Male1	Mana	9	0.091932
1**	Mana	10	0.092288
1**M	Mana	10	0.093062
Fergy	Mana	6	0.093467
1**F	Mana	10	0.094241
Kerin	Mana	2	0.094304
Male2	Mana	9	0.094995
Hockey	Mana	9	0.098675
Hockey	Mana	4	0.098675
Vundu	Mana	9	0.09895
Hockey	Mana	4	0.099965
Female1	Mana	9	0.099977
Crochet	Mana	3	0.100551
Trey	Mana	9	0.102256
Ringo	Mana	12	0.102363
Wicket	Mana	3	0.105018
Victorio	Mana	5	0.105209
Nyami	Mana	10	0.105242
Shadow	Mana	9	0.10525
Janet	Mana	9	0.10532
Oar	Mana	6	0.106559
Amos	Mana	6	0.106745
Opaq	Mana	8	0.107816
Patrick	Mana	9	0.109333
Paintedwolf	Mana	11	0.110528
Tornado	Mana	9	0.110572
Bracket	Mana	9	0.11105

Bravo	Mana	9	0.111526
Wicket	Mana	8	0.112017
Rachel	Mana	8	0.11358
Male1	Mana	5	0.11376
Wicket	Mana	10	0.113927
Fergy	Mana	3	0.114235
Player	Mana	9	0.114674
Snowdrops	Mana	8	0.117346
Mark	Mana	2	0.117438
Dart	Mana	9	0.119157
Tunn	Mana	7	0.121105
Ecko	Mana	6	0.121874
Ecko	Mana	9	0.122995
Taurai	Mana	9	0.123343
Spotted	Mana	7	0.123739
Adam	Mana	7	0.124121
2**	Mana	10	0.124229
Wave	Mana	12	0.124357
Blacktip	Mana	11	0.124414
Cable	Mana	4	0.124506
Dopio	Mana	9	0.12507
1**M	Mana	1	0.125346
4**	Mana	10	0.126849
Nicp	Mana	7	0.126916
Cisor	Mana	9	0.128484
Redcloud	Mana	9	0.128685
Harpy	Mana	9	0.128919
Gena	Mana	9	0.129511
Martel	Mana	9	0.129698
Cochise	Mana	9	0.129796
Chewore	Mana	10	0.132298
Eclipse	Mana	9	0.132911
Oar	Mana	12	0.132987
Sarge	Mana	6	0.134097
Tait	Mana	9	0.134255
Tait	Mana	11	0.134807
Chute	Mana	9	0.135187
Flamelily	Mana	2	0.135692
Vulture	Mana	4	0.13584
Ringer	Mana	8	0.135848
Cable	Mana	5	0.136504
Player	Mana	5	0.136737
Rukomechi	Mana	12	0.137013
Blacktip	Mana	11	0.137743
Jigsaw	Mana	9	0.138375
Topi	Mana	8	0.138695

Blacktip	Mana	9	0.139203
Midnight	Mana	9	0.139216
Trickle	Mana	9	0.139685
Sali	Mana	9	0.140026
Crochet	Mana	3	0.140551
Hockey	Mana	1	0.140551
Ace	Mana	10	0.140857
Skop	Mana	8	0.141415
Clown	Mana	9	0.141529
Fleck	Mana	9	0.142472
Vulture	Mana	7	0.142577
Skop	Mana	2	0.143426
Victor	Mana	8	0.14446
Wave	Mana	11	0.144676
Hornet	Mana	10	0.145221
Knight	Mana	10	0.145415
Ringer	Mana	4	0.145896
Loco	Mana	10	0.145993
Scoop	Mana	12	0.146185
Scoop	Mana	10	0.146187
Flint	Mana	4	0.150361
Hockey	Mana	8	0.152944
Taurai	Mana	3	0.153648
Edna	Mana	9	0.153756
Notch	Mana	7	0.153789
Tylor	Mana	9	0.154531
Sandra	Mana	9	0.154682
Viva	Mana	9	0.154682
Janet	Mana	9	0.154818
Crochet	Mana	9	0.155756
Cirrus	Mana	9	0.156197
Manta	Mana	11	0.156325
Nyakasanga	Mana	9	0.157242
Linda	Mana	9	0.157997
Eclipse	Mana	5	0.158321
Levi	Mana	9	0.158376
Fiver	Mana	1	0.158478
Mareken	Mana	9	0.159097
Fiver	Mana	11	0.159477
Ringo	Mana	11	0.159672
Adam	Mana	8	0.160672
Adam	Mana	8	0.16121
Blondie	Mana	11	0.162376
Fergy	Mana	11	0.163007
Blondie	Mana	11	0.163036
Tait	Mana	10	0.16412

Ringer	Mana	2	0.166848
Cirrus	Mana	6	0.166958
Snowdrops	Mana	4	0.167186
Hockey	Mana	9	0.168597
Opaq	Mana	9	0.16943
Patrick	Mana	8	0.171407
Petra	Mana	11	0.174936
Cuba	Mana	9	0.177064
Blacktip	Mana	9	0.177325
Mark	Mana	9	0.177938
Cochise	Mana	10	0.179449
Redcloud	Mana	10	0.180745
Obert	Mana	1	0.183457
Snowdrop	Mana	10	0.183518
Fergy	Mana	8	0.184293
Toy	Mana	9	0.185265
Nyami	Mana	8	0.18638
Alert	Mana	9	0.18682
Anna	Mana	9	0.187008
Obert	Mana	9	0.187087
Felix	Mana	9	0.18786
Crochet	Mana	11	0.189747
Opaq	Mana	9	0.190236
Ace	Mana	9	0.193929
Bhoza	Mana	9	0.196282
Alert	Mana	11	0.19667
Opaq	Mana	9	0.2011
Flame	Mana	8	0.201432
Flamelily	Mana	8	0.203809
Spotted	Mana	6	0.204872
Oar	Mana	10	0.211266
Nyakasanga	Mana	9	0.213081
Sandra	Mana	11	0.223237
Ringo	Mana	11	0.226847
