# Supplementary material for: Quantitative photogrammetric methodology for measuring mammalian belly score in the painted dog
Source: PLoS One. 2021 Dec 14;16(12):e0261171. doi: 10.1371/journal.pone.0261171 (PMC8670687; doi:10.1371/journal.pone.0261171)
Supplement: S2 Data — (PDF) [file pone.0261171.s002.pdf]

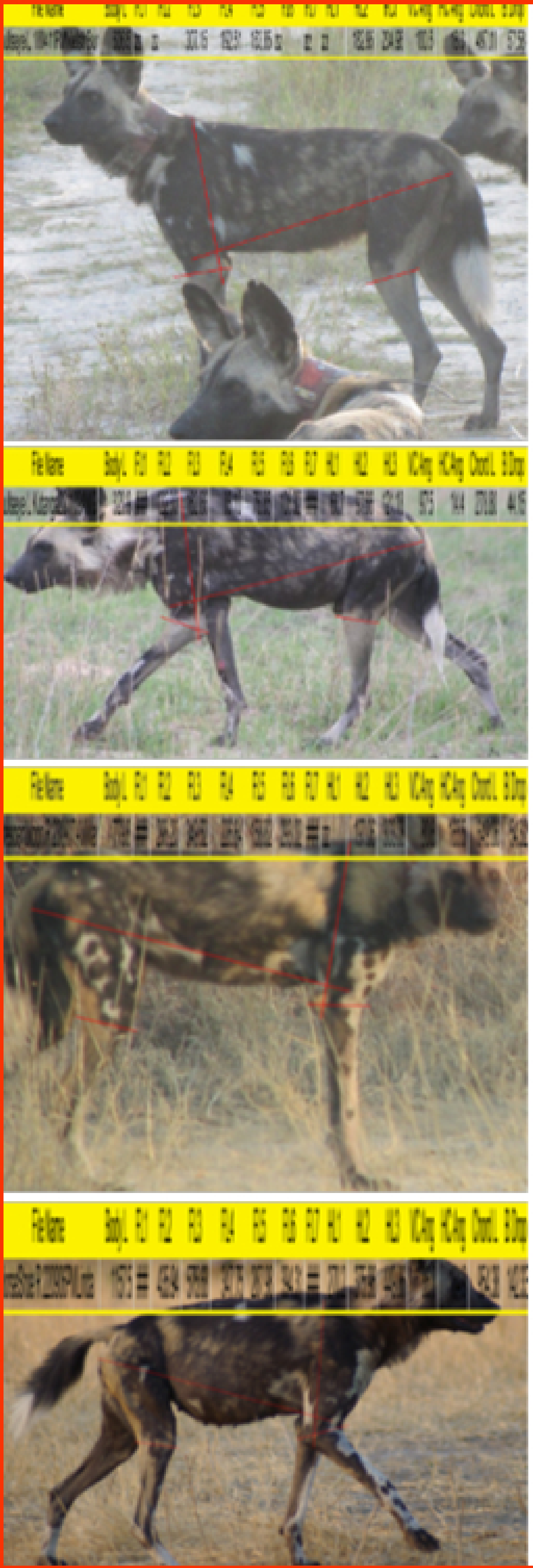

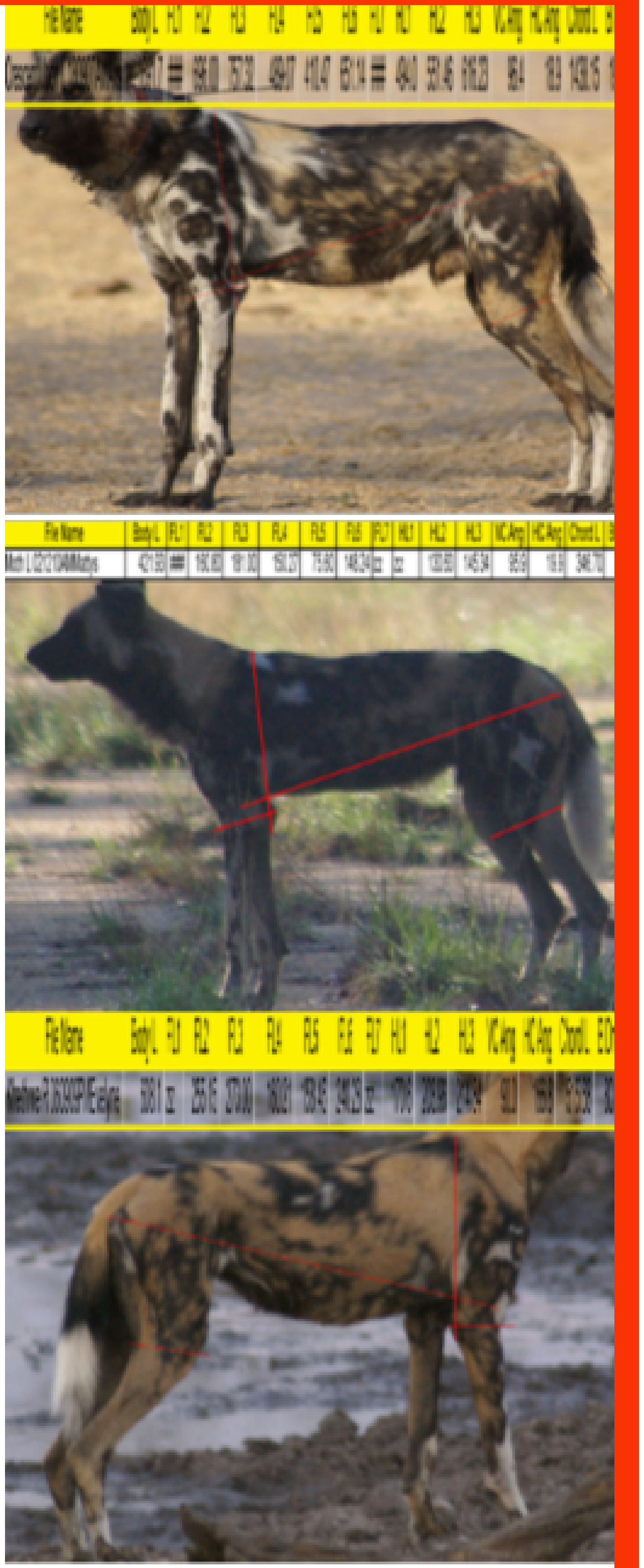

Examples of data images from Hwange

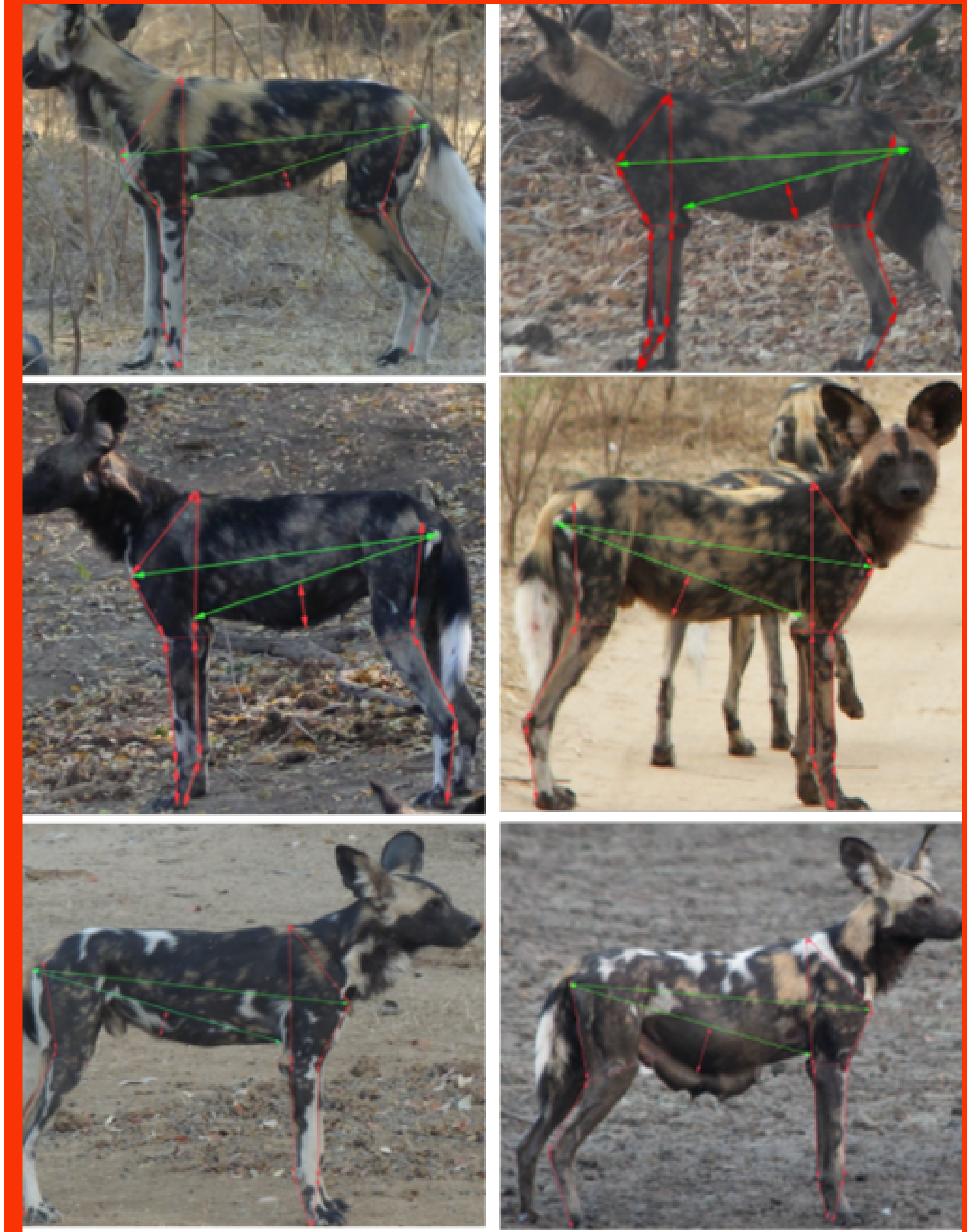

Examples of data images from Mana
